# See one, do one, teach one: Reimagining reproductive endocrinology and infertility training programs to expand access to care

**DOI:** 10.1016/j.xfre.2021.10.001

**Published:** 2021-10-07

**Authors:** Jacob P. Christ, Michelle Vu, Holly Mehr, Tia Jackson-Bey, Christopher N. Herndon

**Affiliations:** a Division of Reproductive Endocrinology and Infertility, Department of Obstetrics and Gynecology, University of Washington School of Medicine, Seattle, Washington; b Department of Obstetrics and Gynecology, University of Rochester Medical Center, Rochester, New York; c Division of Reproductive Endocrinology and Infertility, Department of Obstetrics and Gynecology, University of California Los Angeles, Los Angeles, California; d Division of Reproductive Endocrinology and Infertility, Department of Obstetrics and Gynecology and Reproductive Science, Mount Sinai School of Medicine, New York, New York

**Keywords:** Infertility, access to care, racial inequities, education, immigrant

## Abstract

**Objective:**

To provide a review of the current literature surrounding barriers to reproductive medicine and present examples of how resident and fellow education can be used to overcome these barriers.

**Design:**

A review of the relevant literature addressing barriers to reproductive medicine, resident and fellow education, and related materials was completed.

**Setting:**

Academic medical institutions.

**Patient(s):**

None.

**Intervention(s):**

None.

**Main Outcome Measure(s):**

Health disparities and barriers in access to care.

**Result(s):**

Of barriers in access to care, 3 were reviewed in detail: cost of health care, racial inequities, and marginalization of immigrant communities. The suggested strategies to mitigate these barriers include the following: reducing racial inequities through improved diversity within reproductive medicine and through antiracism training, developing opportunities for trainees to engage in advocacy, strengthening reproductive endocrinology and infertility clinical exposure and educational curricula in training programs, inclusion of residents and fellows in clinical care, and improving the accessibility of fertility care through implementing approaches to optimize the management of infertility in challenging, resource-constrained settings.

**Conclusion(s):**

Infertility is one of the most prevalent reproductive health diseases, yet profound disparities and inequities in access to care exist today in the United States. Lower-income, minority, and immigrant communities are among those most marginalized. Improved access to care begins with broadened obstetrics and gynecology and reproductive endocrinology and infertility trainee education, which acknowledges the barriers these communities face and provides strategies to help overcome these obstacles to care.

**Discuss:** You can discuss this article with its authors and other readers at https://www.fertstertdialog.com/posts/xfre-d-21-00113

Infertility affects an estimated 48 million couples and, by some estimates, up to 186 million women worldwide (1). The World Health Organization recognizes infertility as a disease and has ranked infertility as the fifth leading generator of disability among people aged <60 years (2, 3). Globally, less than 5% of people have access to effective infertility care (4). In the United States, approximately 76% of the demand for assisted reproductive technologies (ART) is unmet (5). Infertility and access to infertility are major public health issues in both the United States and worldwide (6, 7).

Postgraduate medical training programs in the United States are centered at academic medical centers, US military medical facilities, community-based hospitals, and ambulatory care centers that often serve as access to care points for diverse and lower-income patient communities. With the proper development of infrastructure and curricula, these centers create an opportunity for obstetrics and gynecology (OB/GYN) and reproductive endocrinology and infertility (REI) trainees to directly participate in and provide care for patients from disadvantaged groups who may otherwise not have access to infertility care. In turn, the resulting clinical experience will better prepare graduates to care for patients in underserved communities, resource-constrained settings, and areas without available subspecialty reproductive endocrinology services. Many training programs in the United States, however, lack the formalized curricular and clinical structure to optimally prepare clinicians for infertility care in these settings. These deficiencies are present in the backdrop of existing heterogeneity and substantial gaps in REI education and clinical training among OB/GYN residency programs (8, 9, 10). Here, we present a review of 3 main barriers in access to infertility care and identify the key interventions in the framework of postgraduate medical education that can improve access to infertility care.

## Barriers to care

### Cost of Health Care

In the United States, only 1 in 4 people can access the care they need to become pregnant (7). The single largest barrier in access to care is out-of-pocket costs (11). The high cost of in vitro fertilization (IVF) in the United States principally reflects the overall costliness of the US health care system rather than uniquely high service costs intrinsic to IVF as a medical intervention (5). For patients without insurance coverage, financial constraints add to the considerable, and often overwhelming, stress and anxiety experienced with infertility. For a vast number without insurance coverage, financial barriers make accessing infertility treatment prohibitive.

Out-of-pocket treatments for IVF in the United States vary by report and geographic location, but estimates are approximately $23,000 per cycle (12). A prospective study in the San Francisco Bay Area of California reported the median cost per person for an IVF treatment cycle to be $24,373 and the median cost per successful IVF treatment to be $61,377, reflecting the common need for more than 1 cycle of IVF to achieve successful pregnancy (13). These high costs, not covered by most health insurances, make the United States one of the least affordable places in the world to undergo ARTs. In the United States, ART is estimated to cost 52.4% of annual disposable income. In comparison, ART costs 5.6% of annual disposable income in Australia and 11.9% of annual disposable income in the United Kingdom (14).

In the United States, insurance mandates make IVF significantly more affordable, improve usage, and broaden access to care (15, 16). One fresh IVF cycle accounts for 52% of an individual’s average disposable income in states without ART insurance mandates, compared with 13% for states with mandates (14). At present, as of 2021, only 19 states have infertility insurance mandates with varying degrees of coverage. The current landscape for insurance coverage for infertility in the United States is changing: 4 states (Colorado, Delaware, New Hampshire, and New York) have passed legislation for comprehensive coverage in the last 3 years (16). Mandated coverage has been shown to increase ART use by nearly threefold compared with nonmandated states and is associated with lower treatment discontinuation rates (15, 17). Although mandated coverage is the most effective single intervention to address the economic barriers in accessing care, gaps remain even in states with comprehensive infertility insurance mandates. State mandates do not apply, for example, to self-insured health plans. Many persons are also excluded because of stipulations in their health plans and through circuitous and noninclusive rules on the definition of infertility. Coverage for infertility is also generally not included in public health programs, such as Medicaid (18). Although not all encompassing, mandated insurance coverage is a significant first step toward achieving broader ART use and equity in access to care and should receive continued focus and advocacy from clinicians and government leaders. In the absence of insurance coverage, infertility care is accessible only to those who can afford it, predominantly those who are older, wealthier, White or work for the “right” employer.

### Racial Inequities in Infertility Care

**Figure 1:**
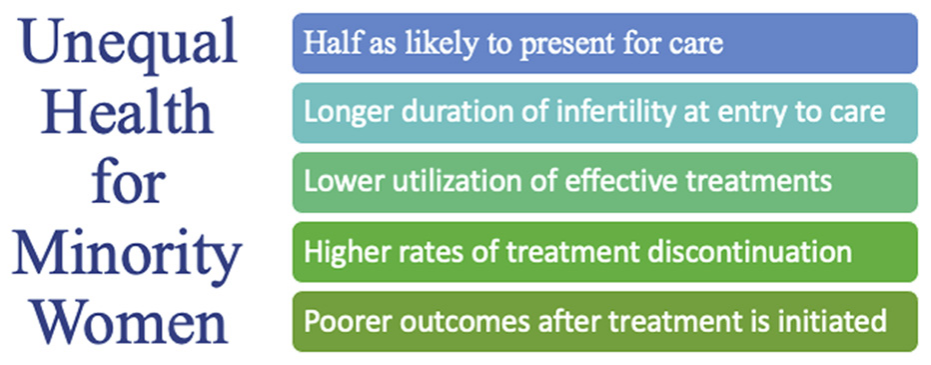
Disparities in infertility health care among minority patients. Minority patients face inequities in access to infertility care, differences in infertility management and resource utilization, and poorer fertility outcomes.

On the basis of the most recent data from the National Health and Nutrition Examination Survey, the rates of infertility between races are overall similar. However, compared with non-Hispanic White and non-Hispanic Asian women, non-Hispanic Black and Mexican/American women saw a medical provider for infertility care approximately half as often (19). Furthermore, Black and Asian women have been found to present with a longer duration of infertility before seeking treatment (18, 20). There is also evidence of poorer fertility treatment outcomes among minority women (21, 22, 23), and among those who seek treatment, there are higher rates of treatment discontinuation (24) (Fig. 1). Racial disparities in the use of ART services can be found even with insurance coverage. This suggests the presence of social and cultural determinants of health beyond the cost of care of which discrimination and unconscious bias cannot be excluded (20, 25). Health care practitioners must evaluate not only the financial and structural barriers to accessing care but also how individual and institutional practices affect outcomes for minority patients.

Racial and ethnic diversity in the medical workforce improves health care quality and access to care and is significant for medical education (26, 27, 28, 29). Diversity of physicians within reproductive medicine, and academic medicine in general, continues to be poor despite calls to increase the recruitment of underrepresented in medicine groups (30, 31). In 2014, 76% of REI providers were Caucasian according to the Society for Reproductive Endocrinology and Infertility Workforce Survey (32). As of December 2018, of 156 REI fellows, only 11 (7%) identified as Black/African American, 22 as Asian (14.1%), 5 as Hispanic/Latinx (3.2%), and 6 (3.8%) as multiracial (30). Unfortunately, there is a paucity of data available to assess the current racial diversity of practicing reproductive endocrinologists and infertility specialists as well as other health professionals in reproductive medicine. There is a pressing need for both increased research on the scope of access to care and health disparities within reproductive medicine and active efforts to increase representation within this field. Fundamentally, access to care is about achieving equity.

### Marginalization of Immigrant Communities

Immigrant patients face unique obstacles to infertility care in the United States, in addition to the previously discussed barriers (33). The immigrant population in the United States is rapidly growing with an estimated 44.9 million immigrants living in the United States in 2019 (34). Immigrants are vulnerable to increased rates of poverty as well as housing insecurity. Furthermore, legal status, fear of deportation, language barriers, and reduced health literacy result in lower rates of insurance coverage and reduced access to preventative health services (35). Infertility assessments and treatments are rarely covered by insurance for this community. If undocumented, immigrants are excluded from Medicaid coverage, and, even if eligible, public health programs typically do not cover infertility care, even in mandated states (18).

Although studies are limited, the experience of infertility among immigrant populations in the United States may mirror that of infertility in the developing world (36). The effects of infertility are often more pronounced in these settings and can lead to profound social consequences. The World Health Organization has ranked infertility as the fifth leading generator of disability among the population of all people aged <60 years worldwide (37). In cultures where children are an integral part of family economic survival and support of elders, infertility can lead to abandonment, seclusion, and domestic violence (38). Furthermore, women are disproportionately blamed and stigmatized for fertility issues (38). The lack of attention to the disparities that immigrant patients experience implies that infertility is a condition undeserving of assistance and minimizes both its impact and importance to patients (3, 39).

## Strategies to improve barriers in access to care among training programs

The first step in attempting to ameliorate disparities is an increased focus on education and increasing awareness of barriers in access to care. In many communities, academic and other training centers serve as an access point for medical care to marginalized and vulnerable populations; indeed, this commitment to equity is often underscored in hospital and university mandates. These institutions are, thus, uniquely positioned to expand access to infertility care, particularly to disadvantaged populations for whom health care outreach is challenging. We propose that infertility care for racially diverse and low-resource populations be included as a key tenet of OB/GYN resident and REI fellow training. We present a summary of these strategies as they relate to racial inequities in infertility care, advocacy, and education and provide suggestions on how to incorporate residents and fellows into reduced cost fertility care.

### Racial Inequities in Reproductive Medicine Training

The past year has held a spotlight to the several ways the medical community has exacerbated health care disparities through systemic racism as well as explicit and implicit bias. Efforts to improve racial disparities must first include an internal assessment of institutional diversity and equity, including the training of its residents and fellows (40). Thus, starting with recruitment, efforts to improve access to training for residents and fellows from diverse backgrounds should be put in place. Several examples of such strategies have been published including mentorship programs, a reduced focus on screening tools based on scholastic performance, and the use of standardized interviews and diverse representation among interviewers (41, 42, 43, 44). Furthermore, the recruitment of diverse faculty and implementation of institutional efforts that include multifaceted approaches to mentorship and sponsorship are needed (29, 30).

Resident and fellow education programs targeted at improved diversity and inclusion and antiracism efforts are also necessary. Such programs have been found to be effective methods of improving knowledge on diversity and inclusion in academic medicine (45). Antiracism training should also be implemented to reduce health inequities and allow for an evaluation of how systemic racism may be contributing to poor patient outcomes (46). These goals align with and support the 2 main charges of the American Society for Reproductive Medicine 2020 Diversity, Equity and Inclusion Task Force to enhance diversity and equity and inclusion of underrepresented minority populations in the profession and reduce and eventually eliminate disparities in care (30).

### Fertility Advocacy

Advocacy is a powerful tool that can be used to improve access to and reduce inequities in fertility care. Advocacy is defined as the application of information and resources to effect systemic changes that shape the way people in a community live (47). Scientific data provide guidance but are often not sufficient alone to bring about policy changes; governments and organizations tend to enact change in the climate of public readiness (48). Physicians and trainees are uniquely poised to help shape public knowledge and acceptance through advocacy by sharing experiences and engaging in organized advocacy efforts (49). RESOLVE: The National Infertility Association hosts, since 1994, an annual Federal Advocacy Day where participants are trained in how to be an advocate and then meet their national legislators to lobby for improved access to fertility care. Social media also provides an emerging avenue for virtual advocacy, which has recently been highlighted by the American Society for Reproductive Medicine Committee Opinion on the use of social media in reproductive medicine (50). The use of social media by reproductive medicine groups has become commonplace, with over 3 million posts related to infertility on the social network Instagram in April 2019 alone (40). Social media platforms could, thus, facilitate broad civic engagement and collective action when applied for advocacy efforts.

Training programs can advance access to care through supporting opportunities and training for advocacy. Although perhaps underappreciated, the Accreditation Council for Graduate Medical Education and Council on Resident Education in OB/GYN explicitly include advocacy as a curricular educational component (51, 52). Physicians’ professional responsibilities extend beyond clinical practice and include advocacy to promote societal health and well-being. As respected leaders in the community, clinicians can share their firsthand experiences and medical knowledge, allowing them to be impactful health care advocates. Unfortunately, this role can be easily lost between the competing needs of the physician and patient and availability of health care resources without robust institutional support (53). The inclusion of formal curricula on advocacy training would help ensure that, despite these competing interests, physicians have the tools to advocate for their patients.

### Education

**Figure 2:**
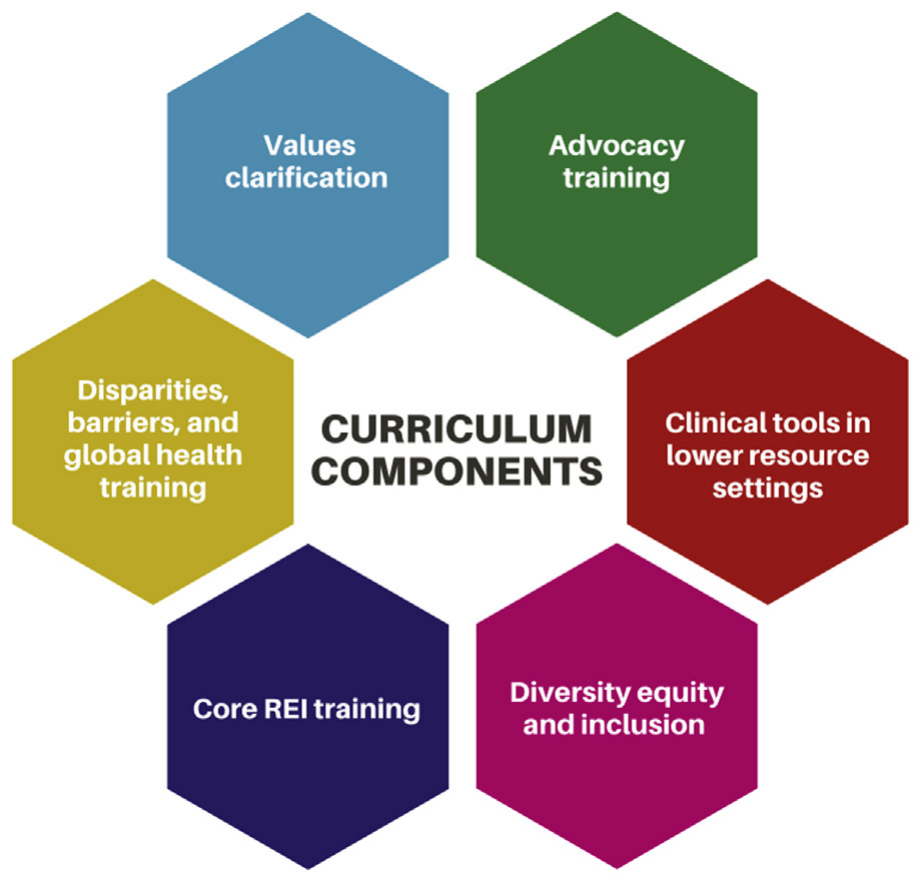
Key curriculum elements for REI training programs with the goals of improving training in REI and broadening access to care. REI = reproductive endocrinology and infertility.

Approximately 40% of reproductive-aged women do not have nearby geographic access to a comprehensive ART center (54). An increased focus on REI training for OB/GYN residents can help reduce geographic barriers to care by allowing for broader and greater inclusion of infertility care into the scope of practice of specialist OB/GYN physicians and women’s health practitioners. We propose a comprehensive curriculum (Fig. 2) aimed at broadening access to care that would operate within 3 domains of learning: the clinical aspect of infertility care in resource-limited settings (cognitive domain); trainee concerns and comfort in the provision of infertility to lower-income and minority individuals (affective domain); and skills development through clinical exposure to the management of complex patient populations with several barriers (psychomotor domain) (55).

Large gaps in knowledge about reproductive medicine have been identified among OB/GYN residents even though infertility is included in the core learning objectives of OB/GYN residency programs (8, 9, 10, 52). Although most graduates understand available ART treatments, success rates for ART are often overestimated, and there is a lack of knowledge surrounding age-related fertility declines (9, 10, 56). Improved REI clinical training during residency would allow graduates to feel more comfortable and proficient in the initial diagnostic evaluation and management of infertility. This would result in a wider availability of fertility services as several forms of infertility do not require ART. Reproductive endocrinology and infertility curricula should also include the psychosocial impact of infertility, health disparities, barriers in access to care, and biases training. Further, infertility should be taught in a global health and cross-cultural perspective in context of its prevalence, impact, untreated disease burden, and considerations relevant to providing clinical care to international patients (2, 3).

Infertility is often overlooked in discussions of reproductive justice. Historically, little attention by medical and public health organizations has focused on making infertility care available to a wider population. Far too often, the suffering of infertile patients in resource-limited populations goes unrecognized from a lack of empathy and biases even from health care providers (57). Misguided arguments against providing infertility care include that it is elective, too costly, and too technical and that the focus should be placed on other health conditions (58). There may be false perceptions and implicit bias among health care workers that lower-income and immigrant patients do not struggle with infertility, supporting infertility treatments will result in children whom are unable to be cared for, and families will require social support programs or even will contribute to problems of overpopulation (33, 38). Same-sex couples and unpartnered individuals can also be denied care by religious-affiliated health care institutions and others (59). Refusing to provide or support infertility care to these groups fails to recognize the immense personal, social, financial, and societal implications infertility has in these settings (38). Furthermore, it incorrectly assumes that fertility care will require large costs to society (5).

Resident and fellow training should engage and challenge these wrongly held beliefs about infertility and work toward strategies to counteract them. Leaders in family planning have long acknowledged that our biases, values, and assumptions influence how we respond to our patients’ needs and can negatively influence the patient-provider relationship for those seeking abortion counseling. For this reason, family planning training for OB/GYN residents often incorporates values clarification to help reduce the stigma associated with abortion care (60, 61). The use of a similar strategy for infertility training should also be considered to help reduce bias, which likely limits access to care for minority and lower-income groups. Examples include the rights of immigrants, unpartnered individuals, lesbian, gay, bisexual, transgender, and queer/questioning persons, patients with higher medical complexity elevating the risk of pregnancy, persons of advanced reproductive age, and people of lower income to receive infertility care. Values clarification discussions are a significant tool to acknowledge that we possess and act on values and attitudes that may be guided by misinformation and internalized social norms rather than factually correct information or an understanding of how restricting access to health care services increases women’s health risk.

### Inclusion of Residents and Fellows and Optimizing Fertility Practices to Improve Access to Care

Lower resource, racially and culturally diverse settings pose unique challenges and considerations for providers and patients. Major barriers to effective clinical practice have been identified and include accessibility, biases, communication, cost, and efficiency (Table 1) (33, 36, 62).

**Table 1:** Barriers encountered by socioculturally diverse, lower-income patient populations seeking infertility care.

Barrier type	Barriers encountered
Accessibility	• Bureaucracy of health care systems • Legal status of immigrants and fear of deportation • Difficulty accessing infertility specialists • Difficulty scheduling appointments • Transportation • Lack of childcare • Lack of social support
Biases	• Racism and discrimination • Implicit bias and false perceptions by health care professionals • Lack of diversity in the REI field • Cultural stigmatization of infertility • Deprioritization of infertility by health care systems • Patient distrust of health care institutions
Communication	• Language barriers • Availability of interpreters • Health literacy • Lack of language and literacy appropriate educational materials • Lack of cultural understanding by providers
Cost	• High cost of health care • Limited or lack of insurance coverage • Lack of public health insurance coverage • Financial transparency • Inability to qualify for discount programs
Efficiency	• Resource-constrained clinics • Lack of clinic infrastructure for providing infertility care • Onsite availability of diagnostic testing (e.g., SA, HSG) • Lack of streamlined protocols for diagnostic evaluation and treatment • Lack of referral pathways for subspecialty care and ART • Lack of provider continuity • Delays in follow-up

*Note:* Primary barriers to infertility care among minority, immigrant, and lower resource patients. ART = assisted reproductive technologies; HSG = hysterosalpingogram; REI = reproductive endocrinology and infertility; SA = semen analysis.

Low technology solutions can be adopted to attempt to reduce these barriers and improve access to care. For example, limited English proficiency has been linked to increased adverse events in US hospitals (63). Although in-person interpreters are generally preferred by patients and providers, using telephonic or video interpreters during clinical encounters is effective and helps reduce patient wait times (64). Furthermore, access to straightforward information pamphlets written at grade 6–7 level and translated into common languages can help provide basic infertility information to patients in low-resource settings who often have a lower level of education and understanding of infertility (36, 65, 66). These educational materials can be printed materials or, through the production of short videos, posted online. Other basic strategies include the development of streamlined protocols to allow for consistency between providers, attempts to consolidate evaluations and clinic visits, providing transportation resources and reimbursement for visits, and education and integration of specialist gynecologists into fertility care.

The incorporation of residents and fellows into infertility practices can also help improve accessibility, aid with cost reductions, and provide opportunities for improved patient continuity, “hands-on” patient care, graduated clinical responsibilities, and exposure to infertility management across diverse sociocultural demographics. Successful examples of programs include the low-cost and complexity IVF program at the University of California, San Francisco (UCSF) for patients at San Francisco General Hospital and the resident continuity clinic program at New York University’s Bellevue Hospital (67, 68). In the UCSF model, basic diagnostic evaluations are completed at a weekly OB/GYN resident REI clinic at San Francisco General Hospital, the public teaching hospital affiliated with the UCSF that provides care to patients from socioculturally diverse, less affluent, and largely immigrant communities. Patients needing IVF are referred to a reduced cost and simplified IVF program operated by UCSF REI fellows under the preceptorship of REI faculty attendings (3). At Bellevue Hospital, initial health screenings, laboratory evaluations, and ovarian reserve testing are completed by a gynecology resident. A patient is then referred to REI as appropriate, and initial REI evaluations and treatment such as ovulation induction are completed by a resident or fellow with attending supervision (68).

Resident exposure to REI training varies widely by geographic region because most REI fellowship programs are located in urban centers in the coastal United States (69). Even in the absence of an onsite REI clinical practice, resident continuity clinic practices can develop evidence-based protocols for initial infertility diagnostics (including site-specific guidance on where and how to obtain semen analysis and hysterosalpingogram), ovulation induction for polycystic ovary syndrome, as well as referral pathways for subspecialty care as indicated.

Protocols proposed to reduce ART cost, including mild stimulation, intravaginal incubation, and simplified laboratory handling, may be used to augment efforts to improve insurance coverage for IVF, as a means to broaden access to care. In natural cycle protocols, a single oocyte is retrieved using either no (natural cycle) or minimal (modified natural cycle) medication, and in mild stimulation protocols, 2–7 oocytes are retrieved using letrozole or clomiphene citrate with or without reduced doses of gonadotropins (70, 71). The application of mild stimulation protocols in a low-resource population and in a primarily fellow operated setting has been shown to result in clinically significant pregnancy rates at significantly reduced costs (3). Additionally, intravaginal culture is a Food and Drug Administration–approved alternative to the use of traditional embryology laboratory incubators, which could, in some settings, lower costs; however, outcome data for this method are more limited (72, 73, 74). These and other strategies can be implemented by clinical programs operated by residents and/or fellows to help lower financial barriers for patients.

### Summary and Recommendations

The majority of people in the United States are unable to access adequate fertility care. Several barriers to care exist that disproportionally affect low-resource, minority, and immigrant communities. The single largest barrier to care remains the cost of medical care. Only 19 states currently have some extent of mandated insurance coverage for infertility care and broader coverage is needed. Racial disparities among those pursuing fertility treatments result in reduced access and poorer outcomes for minority groups. Additionally, immigrant communities face unique barriers to care that need increased attention and efforts to overcome.

**Figure 3:**
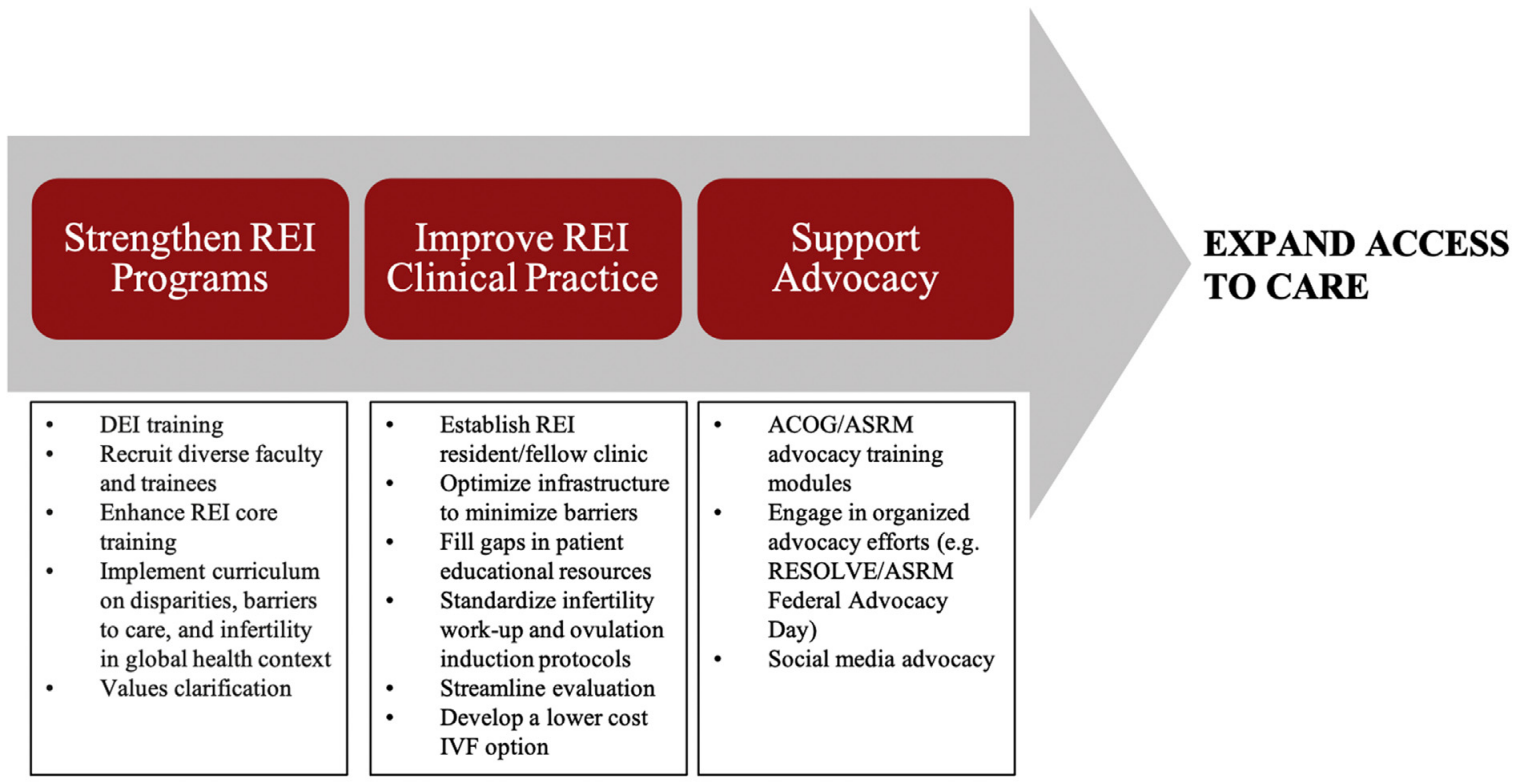
Proposed road map for obstetrics and gynecology and reproductive endocrinology and infertility training programs to expand access to care. ACOG = American College of Obstetricians and Gynecologists; ASRM = American Society for Reproductive Medicine; DEI = diversity, equity, and inclusion; IVF = in vitro fertilization; REI = reproductive endocrinology and infertility.

We propose that resident and fellow training, centered at academic centers, should be a key tenet for improving access to care. We outline a proposed strategic road map to realize this change (Fig. 3). A first step toward reducing racial inequities includes increased diversity within REI physicians, trainees, and staff as well as education programs targeted at improved equity and antiracism efforts. We encourage resident and fellow advocacy to work toward expanded mandated coverage as well as broader inclusion for those who qualify for insurance coverage. An increased focus on REI within OB/GYN residencies, which includes values clarification training, will allow OB/GYN physicians to feel comfortable providing the initial workup and management for infertility and will reduce the stigma associated with infertility care. Finally, residents and fellows should be integrated within REI clinical practices, which incorporate reduced cost ART strategies to both improve training and provide broader access to care for lower-income patients.

In conclusion, OB/GYN and REI training that promotes diversity and equity, integrates advocacy, includes values clarification, and broadens clinical exposure for trainees is a central component to reducing the significant barriers to care that lower income, minority, and immigrant populations face.
